# The Biological Diversity and Production of Volatile Organic Compounds by Stem-Inhabiting Endophytic Fungi of Ecuador

**DOI:** 10.3390/jof1030384

**Published:** 2015-12-02

**Authors:** Susan M. Rundell, Daniel J. Spakowicz, Alexandra Narváez-Trujillo, Scott A. Strobel

**Affiliations:** 1 Department of Ecology and Evolutionary Biology, Yale University, New Haven, CT 06520, USA; E-Mail: susan.rundell@yale.edu; 2 Department of Molecular Biophysics and Biochemistry, Yale University, New Haven, CT 06520, USA; E-Mail: daniel.spakowicz@yale.edu; 3 Laboratorio de Biotecnología Vegetal, Pontificia Universidad Católica del Ecuador, Quito 17 01 21 84, Ecuador; E-Mail: anarvaez@puce.edu.ec

**Keywords:** biodiversity, *Diaporthales*, endophytic fungi, monoterpenoids, neotropics, volatile organic compounds

## Abstract

Fungal endophytes colonize every major lineage of land plants without causing apparent harm to their hosts. Despite their production of interesting and potentially novel compounds, endophytes—particularly those inhabiting stem tissues—are still a vastly underexplored component of microbial diversity. In this study, we explored the diversity of over 1500 fungal endophyte isolates collected from three Ecuadorian ecosystems: lowland tropical forest, cloud forest, and coastal dry forest. We sought to determine whether Ecuador’s fungal endophytes are hyperdiverse, and whether that biological diversity is reflected in the endophytes’ chemical diversity. To assess this chemical diversity, we analyzed a subset of isolates for their production of volatile organic compounds (VOCs), a representative class of natural products. This study yielded a total of 1526 fungal ITS sequences comprising some 315 operational taxonomic units (OTUs), resulting in a non-asymptotic OTU accumulation curve and characterized by a Fisher’s α of 120 and a Shannon Diversity score of 7.56. These figures suggest that the Ecuadorian endophytes are hyperdiverse. Furthermore, the 113 isolates screened for VOCs produced more than 140 unique compounds. These results present a mere snapshot of the remarkable biological and chemical diversity of stem-inhabiting endophytic fungi from a single neotropical country.

## 1. Introduction

Endophytes are a diverse array of organisms—including fungi and bacteria—that are capable, for at least part of their life cycles, of living within the tissues of living plants without causing any apparent harm to their hosts [1]. All healthy plants collected from natural ecosystems have been found to harbor at least one endophytic fungus [2]. These endophytes form relationships with their host plants ranging in nature from mutualism to saprophytism [3].

Several studies have been conducted surveying the diversity of fungal endophytes within various ecosystems. Arnold and Lutzoni [4] found a pattern whereby the diversity of foliar endophytic fungi increased with decreasing latitude. They evaluated the diversity of fungal endophytes colonizing the leaves of common plants in arctic, boreal, temperate, and tropical regions, and found the greatest diversity at Barro Colorado Island, Panama [4]. An earlier study on Barro Colorado Island found that residing within 19 trees of two co-occurring species were a total of 242 morphospecies of fungal endophytes [5]. This is likely an underestimate of the true number of morphospecies colonizing these two species of plants alone, given that researchers only counted those endophytes that were readily culturable. When accounting for the massive diversity believed to be characteristic of endophytic fungi, especially in the tropics, the estimate of 1.5 million fungal species in use since the 1990s could be an underestimation [5]. As endophytes have received increasing attention, revised estimates have predicted nearly as many fungal endophytes as was once predicted for the entire fungal richness of the world [6,7].

The most extensive studies to date, however, have focused mainly on foliar endophytes, while stem-inhabiting fungal endophytes have remained comparatively understudied [8]. One group of researchers evaluated the diversity of endophytes inhabiting trees non-native to Argentina in a nature reserve within that country [8]. That group, however, only sampled the bark of three tree species, and recovered 57 fungal isolates. A similar study was conducted using the xylem of healthy Chilean trees, but it, too, surveyed only a handful of tree species, and researchers recovered fungal endophytes from only 51 of 100 samples [9]. Large-scale surveys of stem- or wood-inhabiting fungal endophytes analogous to those carried out by Arnold and Lutzoni [4] for foliar endophytes are currently absent from the literature. Such a study will be necessary to develop a more complete picture of endophytic—and fungal—biodiversity.

One consequence of the wide diversity and unique ecology of endophytes is their potential to produce a wide variety of natural products [10]. Endophytic fungi have increasingly become targets in the search for potentially novel secondary metabolites, in part due to the singular interactions, pressures, and stresses faced by organisms inhabiting this niche [11]. Due to the fact that this class of fungi is understudied, there is little doubt that endophytes represent a largely untapped source of biodiversity, and the novelty and diversity that characterizes the biology of these microbes is likely reflected in the chemistry of their natural products [11,12].

Volatile organic compounds (VOCs) comprise part of an organism’s “metabolome,” providing a useful indication of chemical diversity as a representative class of natural products [13]. It is thought that VOCs help mediate relationships between fungi and other organisms—such as host plants in the case of endophytes—and some 250 VOCs have been demonstrated to be produced by fungal endophytes [14]. Endophytic VOCs are proposed for potential uses ranging from biocontrol agents, antibiotics, commodity chemicals, and biofuels [11,14,15,16]. Unlike other fungal products, they have the advantage of being easily identifiable and quantifiable without extraction. They are therefore ideal candidates to survey the diverse range of natural products made by endophytes quickly and efficiently, well suited to the growing field of metabolomics.

The aim of this study is to both assess the diversity of the endophytic isolates collected from Ecuador over seven years, and to characterize one example of the potential that these endophytes have to generate a diverse array of natural products. This paper details the α diversity represented in 1526 sequenced fungal isolates from Ecuador, as well as the VOC profiles of a subset of those isolates. Our results indicate that Ecuadorian endophytic fungi are characterized by extreme diversity that our sampling, extensive as it was, could not adequately capture. These data demonstrate not only the biological diversity of Ecuadorian endophytes, but also their prodigious chemical diversity, indicated by the wide range of VOCs produced by a small subset of the country’s mycological richness. The scientific community has only just begun to scratch the surface of the biological and chemical wealth that is represented by Ecuador’s endophytic fungi.

## 2. Materials and Methods

### 2.1. Sampling and Isolation

Plants were sampled from three Ecuadorian ecosystems including the coastal dry forest, in the Cerro Blanco Protected Forest (S 2°10′48′′, W 80°1′16′′), in the cloud forest near Mindo (S 0°0′16′′, W 78°44′57′′), and the rainforest, in the vicinity of the Yasuní Research Station (S 0°40′17′′, W 76°24′1.8′′). Each of over 100 students collected healthy stem segments from between twenty and thirty plant specimens representing 131 plant families. Stem segments were clipped from mature branches with shears and placed in sealed, airless plastic bags and refrigerated. Within one to two weeks of collection, the stem segments were surface-sterilized after being dipped in 70% ethanol and passed through the flame of a Bunsen burner. The end of each stem was cut off at this stage to prevent plating samples that were already overcome with rot. The outer layers of the stems were removed using flame-sterilized scalpels. Three stem segments, approximately 1 inch each, were plated on three media types—water agar (WA), 1:10 malt agar (MEA), and 1:10 potato dextrose agar (PDA)—for a total of nine stem segments. Dilute media were used in order to reduce the growth rates of all isolates. The plates were sealed and left to grow at room temperature. Plates were monitored for hyphal growth several times a week. Hyphae were transferred first to 1:10 PDA before being isolated in pure culture on 1× PDA.

### 2.2. Endophyte Identification and Diversity

The nuclear ribosomal internal transcribed spacer region (ITS) was used to classify the fungi in this study. The ITS region was preferred for several reasons, including the resolution it provides at both the inter- and intraspecific scale, its relative ease of amplification, and the practicality of using a region favored by many other mycologists for the purposes of phylogenetic placement and analysis [17,18]. To extract isolate DNA, small pieces of fresh mycelial growth were scraped into 50 μL of sterile water, which was then heat-shocked at 94 °C for 5 min. The DNEasy Plant Mini Prep Kit (Qiagen, Hilden, Germany) was used to extract DNA for each sample. The reaction concentration of each reagent was 1× buffer, 2.5 mM MgCl_2_, 200 μM dNTPs, 1.5 μM each of ITS1 and ITS4 primers [19], and 2.5 U of Taq DNA polymerase. From this mixture, 45 μL was added to 5 μL of heat-shocked template and kept on ice prior to PCR. This mixture was run on a thermocycler through the following cycles: one cycle lasting 300 s at 95 °C, thirty cycles lasting 30 s each at 95, 55, and 72 °C, and one cycle lasting 300 s at 72 °C. PCR mixtures from successful reactions were then sent to the Keck DNA Sequencing Facility at Yale University (New Haven, CT, USA). All sequenced endophytes were placed into permanent water stocks, whereby plugs of 1× PDA containing fresh hyphal growth were placed in cryovials with 750 μL of sterile water and refrigerated. Water stocks were subsequently deposited in the Herbarium at the Yale Peabody Museum of Natural History (New Haven, CT, USA) and Endophyte Collection of the QCA Herbarium at the Pontificia Universidad Católica del Ecuador (Quito, Ecuador).

Consensus sequences were then analyzed using QIIME (Quantitative Insights into Microbial Ecology), an open source package used for analyses of and comparisons between communities of microbes such as fungi [20]. This program used ITS sequences from UNITE, a database containing over 350,000 fungal ITS sequences, to assign taxonomic classifications to each isolate [21]. The results of this classification were subsequently visualized using Krona [22]. An operational taxonomic unit (OTU) of 97% was used to assess the richness of the Ecuadorian samples over a range of taxonomic levels [23,24]. This OTU was also used to generate a species accumulation curve, to count singletons, and to estimate Shannon Diversity and Fisher’s α for all sequenced Ecuadorian isolates. Shannon Diversity (*H*) is given by the formula

(1) $$H=-\overset{s}{\underset{i=1}{\sum }}p_{i}\;lnp_{i}$$

where *s* is the species richness and *p_i_* is the proportion of *s* made up by the *i*th species [25]. Fisher’s α is implicitly defined by the formula

(2) $$s=\alpha ln(1\;+\frac{n}{\alpha })$$

where *s* is the species richness and *n* is the sample size [26].

### 2.3. Volatile Organic Compound (VOC) Survey

A total of 113 randomly selected isolates were grown in sealed GCMS (Gas Chromatography-Mass Spectrometry) vials for approximately two weeks on 10 mL 1× PDA. The subsequent analysis was carried out using a GCT Premier gas chromatography time of flight mass spectrometer (GCMS) (Waters, Milford, MA, USA) with a DB-5 column (30 m × 0.25 mm ID × 1 µm film thickness; Agilent, Santa Clara, CA, USA). VOCs in the headspace above the endophytes were sampled with a 50/30 μm divinylbenzene/carboxen/polydimethylsiloxane StableFlex SPME Fiber (Supelco, Bellefonte, PA, USA). The data were analyzed using the MassLynx Software Suite (Waters), and retention indices were generated by comparing compound retention times with those of an alkane mix (Fluka, Buchs, Switzerland). When possible, mass spectra were compared to those of commercially available standards (Sigma-Aldrich, St. Louis, MO, USA). The program jModeltest2 was used to determine the most appropriate nucleotide substitution model for resolving the phylogenetic relationships among these 113 isolates [27,28]. Ultimately, the TrN + I + G model was used to construct a maximum likelihood phylogeny from 1000 replicates in MEGA6, and the resulting consensus tree was subsequently visualized alongside a heat-map of VOC data using Interactive Tree of Life [29,30,31].

## 3. Results

### 3.1. Endophyte Diversity

**Figure 1:**
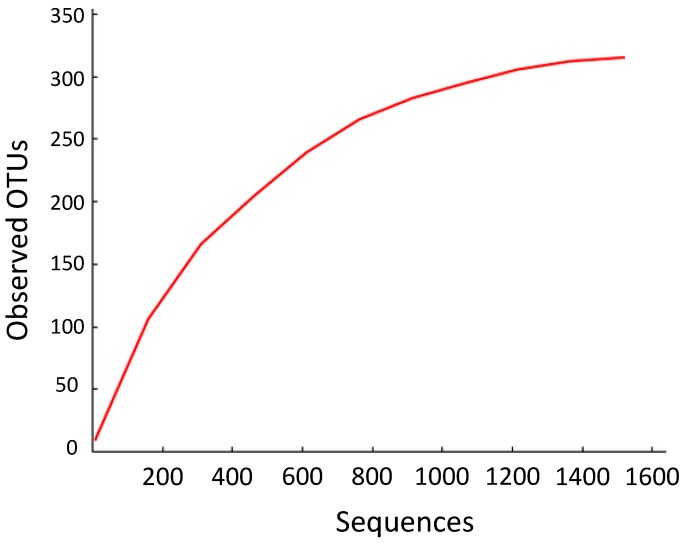
Species accumulation curve using operational taxonomic units (OTUs). Visualized using QIIME (Quantitative Insights into Microbial Ecology) [20].

**Figure 2:**
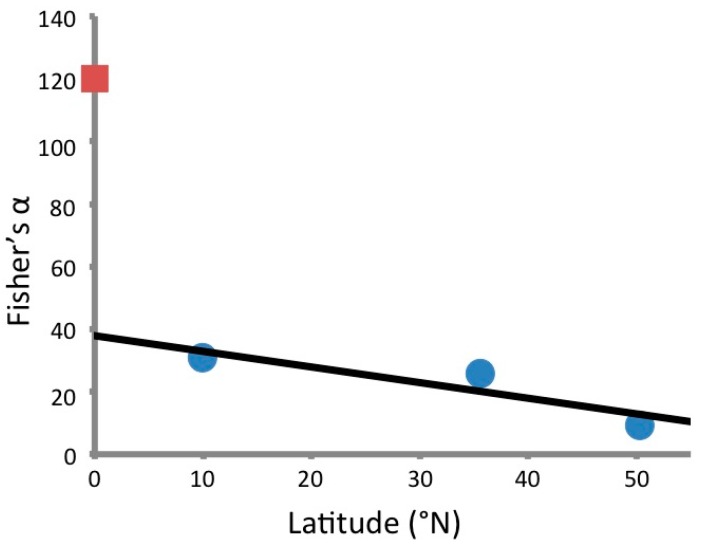
Fisher’s α *versus* latitude of Ecuadorian stem-inhabiting endophytes (red square) and foliar endophytes collected from angiosperms by Arnold and Lutzoni in 2007 [4] (blue circles).

Ultimately, 1526 unique fungal morphotypes were obtained from 861 plated plant specimens (Supplementary Material 1). When sequenced, these isolates represented 315 OTUs (Figure 1). The Fisher’s α for the sequenced fungal endophytes was 120, and the Shannon Diversity index was approximately 7.56. By contrast, a study by Arnold and Lutzoni [4] found that 1403 foliar endophytes isolated from four different latitudinal zones had a Fisher’s α of 103; those endophytes collected from neotropical angiosperms at Barro Colorado Island, Panama, had a Fisher’s α of 31. It appears, therefore, that the Ecuadorian isolates are even more diverse than those considered in this previous study, despite the generally higher diversity expected for foliar compared to stem-inhabiting endophytes [32] (Figure 2).

Despite years of collection and thousands of recovered samples by over 100 students, this collection of endophytic DNA sequences fails to capture the richness of even those fungi that were culturable under the conditions of this study. As shown in Figure 1, the species accumulation curve fails to reach an asymptote. Additional sampling of Ecuadorian isolates, therefore, would produce more species of endophytes than have been found throughout this study.

**Figure 3:**
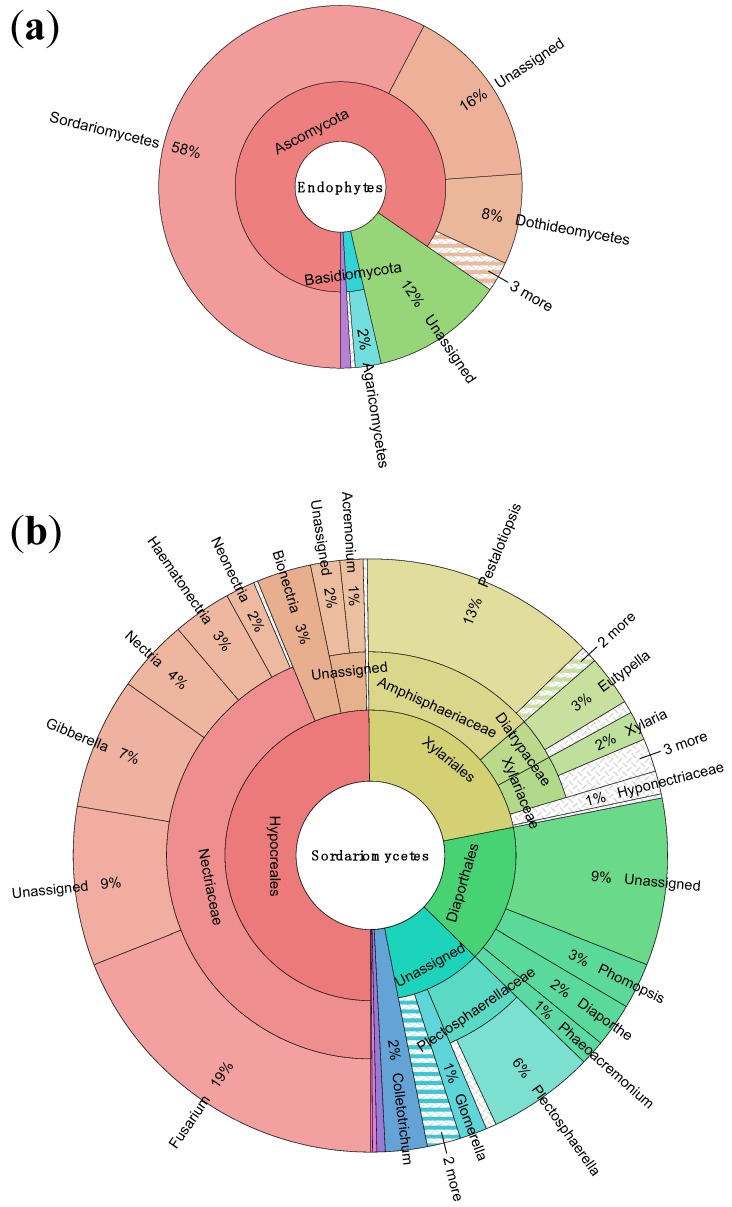
(**a**) Sunburst plot showing taxonomic abundance of 1526 sequences to order; (**b**) Taxonomic abundances within the most abundant order, Sordariomycetes, to genus. Visualized using Krona hierarchical data browser [22].

According to our results, the great diversity of the Ecuadorian endophytes was present at multiple phylogenetic levels. Of 315 OTUs present in the samples, 26 were only observed once, while a further 121 OTUs were observed just twice. Almost 85% of the 1526 sequences were members of the Ascomycota phylum (Figure 3). The most dominant class by far was the Sordariomycetes, which accounted for over 57% of sequences (Figure 3). The three orders that accounted for the most sequences were the Hypocreales, which represented 29% of sequences, followed by the Xylariales, with 13% of sequences, and the Diaporthales, at 9% of sequences. The Nectriaceae family included over 25% of sequences, largely due to the enormous representation of the *Fusarium* genus, which, with its teleomorph *Gibberella*, accounted for over 15% of all sequences. No other single family accounted for more than 8% of isolates.

### 3.2. VOC Production

An analysis of the VOC profiles of 113 endophytes uncovered the production of VOCs with 143 distinct retention times (Supplementary Material 2). A total of 58 distinct sesquiterpenoids were produced. Using a combination of NIST library matches, commercially available standards, and retention indices, a further 68 VOCs were identified to at least molecular formula, including more than a dozen monoterpenoids. Apart from sesquiterpenoids and monoterpenoids, the classes of VOCs produced by the endophytes included: esters, alcohols, ketones, aldehydes, alkanes/alkenes, acids, ethers, ester-acids, and ester-alcohols.

**Figure 4:**
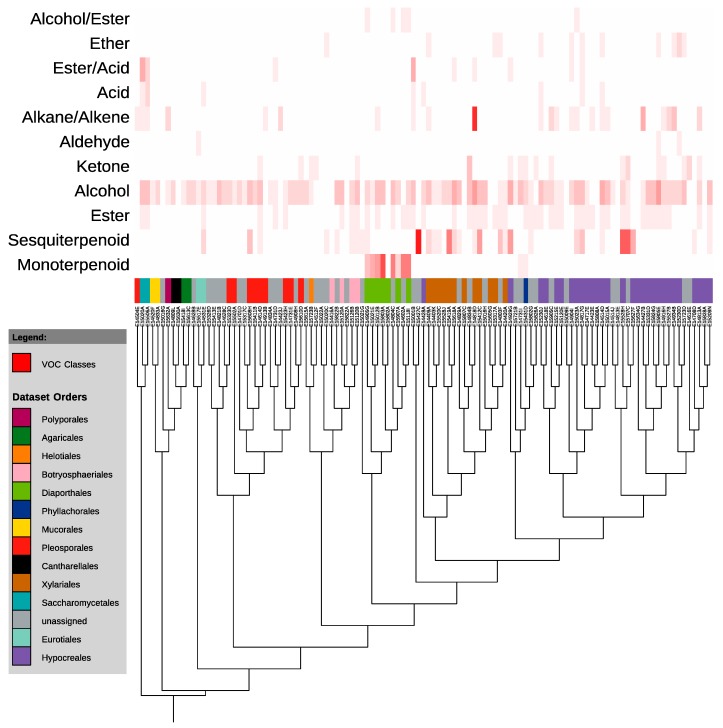
Maximum likelihood tree of 113 isolates analyzed on GCMS (Gas Chromatography-Mass Spectrometry). The heat map on the right indicates the number of compounds of each class produced by each endophyte. Tree was constructed in MEGA6 and visualized using the Interactive Tree of Life [30,31].

According to the overlay of the phylogenetic tree of GCMS-sampled isolates with a heat-map demonstrating the number of compounds of each class produced by each endophyte, VOC production is largely unconnected to evolutionary relationships (Figure 4). There is, however, one exception to this. The monoterpenoids do not appear to be randomly placed throughout the phylogeny (Figure 4). Instead, they appear to be very closely associated with a single clade—the Diaporthales. Specifically, a single genus within the Diaporthales—the *Diaporthe* (telomorph *Phomopsis*)—contains at least 6 of the 10 monoterpenoid-producers of the 113 analyzed, with another two unidentified sequences that likely belong to that same genus. The other two monoterpenoid-producers are in the Glomerellales order, and produce a single monoterpenoid each.

## 4. Discussion

### 4.1. Biodiversity of Ecuadorian Fungal Isolates

It is challenging to put a Fisher’s α of 120 into perspective, especially since other studies of this duration and scale have not previously been conducted for stem-inhabiting fungal endophytes. A 2007 study by Arnold and Lutzoni, however, does offer some means for comparison [4]. In that study, 1403 foliar endophytes were isolated and sequenced from arctic, boreal, temperate, and tropical sites. All together, these 1403 fungi from four latitudinal zones had a Fisher’s α of 103.1 [4]. By this measurement we may therefore conclude that the Ecuadorian collection of endophytes—though taken from a single country in the tropics—is higher in diversity. The Ecuadorian assemblage also has greater diversity, as measured by Fisher’s α, than the tropical site sampled by Arnold and Lutzoni [4] at Barro Colorado Island, Panama. The isolates collected from angiosperms at that site by Arnold and Lutzoni [4] had a Fisher’s α of approximately 31, a figure that—though larger than the values found by the researchers at higher latitudes—is notably less than the diversity of the Ecuadorian isolates. This value is consistent with the pattern of increasing endophytic diversity with decreasing latitude. Located on the equator, it is not unanticipated that Ecuador would have the greatest endophytic diversity studied to date based on the latitudinal pattern investigated by Arnold and Lutzoni [4].

It should be noted that latitude should be interpreted as a correlate rather than a cause of the observed endophytic hyperdiversity, the ecological determinants of which are many. These determinants likely include elevation, climate, floristics, and other environmental conditions beyond the scope of this study. It is known, for example, that floristic diversity in Ecuador is among the highest of any country in the world. A comparison between a 50 ha plot of Barro Colorado Island to a plot of equal size in Yasuní National Park—one of several collection sites included in the present study—demonstrated that the latter contained over three times as many tree species as the former [32]. Given Ecuador’s botanical richness, the corresponding hyperdiversity in endophytic fungi may be expected. The latitudinal gradient in endophytic diversity is merely a single means of predicting the expected diversity of a given location, and the only one presently available [4].

Indeed, the Fisher’s α value for the Ecuadorian isolates is even larger than anticipated based upon previously reported values for higher latitudes [4]. It clearly does not produce a linear fit with the previous endophyte diversity estimates, far exceeding the value predicted by a regression of the data published by Arnold and Lutzoni [4] (Figure 1). It is even more striking given that foliar endophytes are generally more diverse than stem-inhabiting ones [33], though at least one study has demonstrated the opposite relationship [34]. This difference likely reflects both the hyperdiversity of Ecuadorian endophytes and the differences in sampling between the two data sets. The current study was done in three significantly different locations at three different elevations and climates within the host country, and a wide variety of woody vegetation was sampled, representing many growth forms, local environments, and physiological conditions. The Arnold study focused on just the dominant tree species within each location [4]. Furthermore, Fisher’s α at each site was calculated separately for angiosperms and conifers in the 2007 study [4], while we present a single Fisher’s α across all sites and all endophytes regardless of host lineage. The differences in approach makes absolute comparison between the values impossible, but the previous study does provide a sense of scale for our current study. The Fisher’s α measured for sampling in all four locations of the previous study [4] is—at 103—still less than that measured from this one small, but hyperdiverse country.

These figures reinforce the immense diversity of Ecuador’s fungal endophytes. At the same time, the non-asymptotic character of the OTU accumulation data suggest that there is much more left to be discovered in terms of Ecuador’s endophytic biodiversity.

### 4.2. VOC Production in the Context of Biodiversity

Our analysis of volatile organic compound production by fungal endophytes included less than 3% of the endophytes collected over the course of this study, yet these alone produced more than 140 VOCs, 126 of which were identified at least to molecular formula. These VOCs included over 70 potential mono- and sesquiterpenoids, among a wide variety of other compounds including alcohols, acids, and alkanes. While VOCs of the various classes are, for the most part, randomly scattered throughout the phylogenetic tree, at least one class—the monoterpenoids—is tightly connected to phylogeny in this analysis, produced almost exclusively by the *Phomopsis*/*Diaporthe* genus. Indeed, a previous study of volatile production by a *Phomopsis* sp. similarly observed the capacity of an Ecuadorian isolate to produce the monoterpenoid sabinene [35]. Production of compounds such as monoterpenoids by fungal endophytes has attracted particular attention because of the potential use of such compounds as biofuel additives [14,36]. The results of this study suggest that it is possible, at least in the case of VOCs, for single clades of endophytes to be enriched in certain classes of natural products such as monoterpenoids, perhaps in predictable ways.

But this, of course, is an observation from only a single order of fungi. While the *Phomopsis*/*Diaporthe* genus does appear to produce an exceptional number of monoterpenoids, this genus accounts for less than 3% of the total diversity represented by the 1526 sequenced isolates. This observation therefore represents only a single illustration of the potential for these ecologically unique organisms to generate chemically interesting natural products. For just as the isolates included in this study represent a fraction of Ecuador’s endophytic biodiversity, volatile organic compounds comprise just a single class of secondary metabolites.

## 5. Conclusions

Increasingly, microbial diversity in organisms such as endophytic fungi is being recognized as a crucial component of ecosystem biodiversity [37]. These results demonstrate that fungal endophytes add yet another dimension to the diversity of Ecuador’s chemical and biological wealth that remains largely unexplored. These findings are particularly timely in the context of declining biodiversity, which will likely come at the cost of undiscovered and potentially useful natural products that may be lost to humankind before their utility is realized [38]. This study provides another incentive for the exploration and conservation of biodiversity in neotropical forests.

## Supplementary Materials

Supplementary File 1

Supplementary File 2
